# Supplementary material for: Chromothripsis during telomere crisis is independent of NHEJ, and consistent with a replicative origin
Source: Genome Res. 2019 May;29(5):737–49. doi: 10.1101/gr.240705.118 (PMC6499312; doi:10.1101/gr.240705.118)
Supplement: Supplemental Material [file supp_gr.240705.118_Supplemental_file_1.zip › contigs/annotated_contigs/DB91/contig.2.DB91_length_717_mean_cov_5.92887029289.docx]

**DB91_length_717_mean_cov_5.92887029289**

```
CCTATGTTGCCCAGGCTGCTCTCAAACTGCTGACCTCAGGTGATCCACCTGCCTTGGCCTCCTAAGGCCCTGGGGTTACAGGCATGAGC
 >chr17:80371215-80371566 - E=2e-196
CACTGCGCCTGGCCTCTAATTTTTATTATTATTGTTTTTTAACTCATGAATTACTTGGAAATTTAAAAGATTTGGAAATGCGTGGGATT

TTTGGTCCCTTTTCACAACGTTGTGGGTATTTCTCCTGACCCACGCTCGTGTGCACAAATTCTCATCACCAGTTTCTGTCTCCTTCACC

CACCGGCTATGGAAAGGGGCGTCAGAACCTCCACTGAGCATGGACCCAGCAGCGTCTGTGCTTTGGTTGCCACGTGTGTCATGG|TGAT

GCC|CAGGACCAGCCTGGGCAACATGGCAAAACCCTGTCTCTACAAACAATACAAAAATTAGGCAGGTGTGGTAGCACGCGTCTGTAGT
    >chr17:80423303-80423662 - E=3e-204
CCCAGCTGCTCTGGAGGCTACAGGGAGCCTCCAGAGGTGGGAGGATCACCAGAGTCTCCCCACTGAGACTCTCTCCACTGCACTCCAGC

CTGGGTGAGGCCCTGTCTCACAAAGAAAAATATTGTGATCTAATTGCTGCTGGATGACTTGTCCAGAAGCCATGGGGGTGAATGGCAAA

CGCTGAGAAGGCTCCTTGGCTGTCCCCTATGCCACCCACTTGTGCCAGTCAGTGTTTCTTGAGGCCCCTACCTTGCTCTAGCACACTAC

CTACTGT
```
